# The impact of an interprofessional training ward on the development of interprofessional competencies: study protocol of a longitudinal mixed-methods study

**DOI:** 10.1186/s12909-019-1478-1

**Published:** 2019-02-07

**Authors:** Johanna Mink, Anika Mitzkat, André L. Mihaljevic, Birgit Trierweiler-Hauke, Burkhard Götsch, Jochen Schmidt, Katja Krug, Cornelia Mahler

**Affiliations:** 1 Department of General Practice and Health Services Research, University Hospital Heidelberg, Im Neuenheimer Feld 130.3, D-69120 Heidelberg, Germany; 2 Academy of Health Professions Heidelberg, Nursing School, Wieblinger Weg 19, D-69123 Heidelberg, Germany; 3 Department of General, Visceral and Transplantation Surgery, University Hospital Heidelberg, Im Neuenheimer Feld 110, D-69120 Heidelberg, Germany; 4 Department of Nursing Science, University Tübingen, Geissweg 5/1, D-72076 Tuebingen, Germany

**Keywords:** Study protocol, Interprofessional relations, Interprofessional education, Interprofessional collaborative practice, Interprofessional competencies, Interprofessional training ward, Nursing education, Medical education

## Abstract

**Background:**

To meet the patients’ needs and to provide adequate health care, students need to be prepared for interprofessional collaborative practice during their undergraduate education. On interprofessional training wards (IPTW) undergraduates of various health care professions potentially develop a mutual understanding and improve their interprofessional competencies in clinical practice. To enhance collaboration of 6th-year medical students and nursing trainees in the third year of their vocational training an IPTW (**H**eidelberger **I**nter**p**rofessionelle Ausbildungs**sta**tion – HIPSTA) was implemented at the University Hospital Heidelberg, Germany. On HIPSTA future physicians and nurses take care of the patients self responsibly and in close interprofessional collaboration, supervised by facilitators of both professions. Although there are positive experiences with IPTWs internationally, little is known about the impact of IPTW on the acquisition of interprofessional competencies. For future interprofessional training and implementation of IPTWs evaluation of interprofessional learning and collaborative practice on Germany’s first IPTW is of high relevance.

**Methods:**

To evaluate the acquisition of interprofessional competencies the study follows a mixed-methods approach. Quantitative data is collected from undergraduate participants, staff participants and facilitators on HIPSTA (intervention group) and undergraduate participants and staff participants on a comparable ‘conventional’ ward without special interprofessional training (comparison group) immediately pre and post HIPSTA and, as follow-up, after three to six months (T0, T1, T2), using three questionnaires, namely the University of the West of England Interprofessional Questionnaire (UWE-IP), the Interprofessional Socialization and Valuing Scale (ISVS) and the Assessment of Interprofessional Team Collaboration Scale (AITCS). Qualitative data is gathered in form of interviews and focus groups based on semi structured guidelines, video recordings of handovers and overt non-participant observations of daily rounds. Quantitative data will be analysed in a longitudinal comparison, presented descriptively and tested with an analysis of variance. Qualitative data will be analysed deductively and inductively.

**Discussion:**

The results of the evaluation will give insight in undergraduates’, staff’s and facilitators’ experiences and their self-perception of competency development. In addition the results will help identify benefits, challenges and areas for modification when implementing and establishing similar interprofessional training wards.

## Introduction

### Background

Despite the importance of interprofessional collaborative practice (IPCP) in clinical practice in order to maintain high quality patient care [1], undergraduate education of health care professions in Germany as in many other countries is predominantly monoprofessional [2]. It has also been shown that although teamwork, leadership and interprofessional communication and collaboration have repeatedly been claimed as necessary to overcome the challenges within health care [3], these competencies are not or insufficiently addressed and developed within medical and nursing curricula as well as in the curricula of other health care professions [2]. In order to meet future challenges in health care, undergraduate education that is focused on acquirement of knowledge has to be complemented by a competency-based education that enables students to achieve profession specific goals and a patient centred interprofessional collaborative practice.

For improvement of educational strategies to develop interprofessional competencies these need to be identified and described. Within the past eight years a number of frameworks have been developed which describe interprofessional core competencies [4–6]. All frameworks have in common that they describe domains such as interprofessional communication and teamwork, competencies referring to roles and responsibilities, ethical aspects and conflict resolution, as well as reflection and shared decision making as relevant [7]. Interprofessional education seems to be effective during undergraduate education where it plays a vital role in reducing prejudices [8, 9] and has positive effects on interprofessional and professional identity development [10]. However, it has also been shown that the readiness for interprofessional learning can decrease over time [8, 11]. Since experience based learning is a relevant aspect for competency development [12, 13], especially for the development of interprofessional competencies [14], educational strategies are required that give the learners the opportunity not only to learn about interprofessional collaborative practice in a classroom setting but to experience interprofessional collaborative practice in real life [15]. Accordingly learning on an interprofessional training ward (IPTW) is regarded as a good opportunity to acquire interprofessional competencies [16].

In order to enable interprofessional learning and collaborative practice within the undergraduate education of the health professions nursing and medicine, in spring 2017 an interprofessional training ward (IPTW) named “**H**eidelberger **I**nter**p**rofessionelle Ausbildungs**sta**tion” (HIPSTA) was implemented in the Department of General, Visceral and Transplantation Surgery of the University Hospital Heidelberg, being the first of its kind in Germany [17]. It is a two-room section within a surgical ward, where patient care is delivered by medical and nursing undergraduates together and self-responsibly, supervised by facilitators of the respective professions (physicians/nurses). These two IPTW rooms are integrated in a surrounding surgical ward, whose staff is responsible for the HIPSTA patients during the nights and weekends (surrounding ward). For comparison a further ‘conventional’ surgical ward in the same department, without special interprofessional training, is integrated in the study (comparison ward).

Positive outcomes of interprofessional training wards regarding clarification of the own and the others’ professional role as well as the understanding and appreciation of teamwork have mainly been described in Scandinavian countries, where placements on interprofessional training wards have been an integral part of the curricula across the health care programmes for over 20 years [18–24]. Hence, the workplace setting is regarded as an area where interprofessional competencies can be acquired and developed within interprofessional collaborative practice, but still little is known about how these competencies are acquired.

### Research questions

1. How do interprofessional competencies of undergraduates on HIPSTA (intervention group) develop compared to undergraduates on a ‘conventional’ surgical ward (comparison group)?
2. What influence does HIPSTA have on staff working on the surrounding surgical ward HIPSTA is integrated in (intervention group) compared to colleagues on a ‘conventional’ surgical ward, who are not affected by HIPSTA (comparison group)?
3. What influence does HIPSTA have on the interprofessional competencies of the facilitators?
4. What influence does HIPSTA have on the self-perception of the facilitators concerning their teaching competencies?
5. Which basic conditions do facilitators and students (intervention group) describe as beneficial or impeding for an interprofessional education setting?
6. What kinds of competency gain can be observed during the daily interprofessional rounds and handovers?

### Trial design

A prospective mixed methods evaluation is being conducted to systematically gather and assesses initial experiences with the IPTW with self-assessment surveys and observational assessment.

## Methods/design

Aim of this study is to assess the development of interprofessional competencies of the undergraduates and staff on HIPSTA (intervention group) compared to undergraduates and staff on the comparison ward (comparison group) based on quantitative and qualitative self-assessment surveys and observational assessment.

### Study setting

On HIPSTA, a two-room section within a surgical ward at the Department of General, Visceral and Transplantation Surgery of the University Hospital Heidelberg, Germany, four undergraduate medical interns and four nursing trainees (undergraduate participants) are responsible for patient treatment and care, supervised by nursing and medical facilitators. On the comparison ward at the same department ‘conventional’ patient care is delivered by medical and nursing staff and undergraduates without special interprofessional training.

### Study design

To answer research questions 1–3 a prospective quantitative approach with questionnaires will be conducted on HIPSTA (intervention group) and on the ‘conventional’ surgical ward (comparison group). To get an in-depth insight in competency development, beneficial or impeding factors and experiences in general (questions 1–6) interviews and focus groups will be performed with undergraduate participants and facilitators on HIPSTA and with staff participants on the surrounding ward (intervention group), and hand-over video documentations and structured ward round observations will be performed on HIPSTA.

### Interventions

During their four weeks placement on HIPSTA the nursing and medical undergraduates are responsible for pre- and post-surgical inpatient treatment of six patients in two rooms on a visceral-surgical ward, in shift work (early and late shift). They all participate in an introduction day immediately before placement, where they can get to know each other and learn about some relevant aspects of the daily practice on HIPSTA. During the placement they work self responsibly and in close interprofessional collaboration, supervised by two facilitators, one of each profession (nurse / physician). Undergraduate participants work in a tandem (nursing and medical), each tandem being responsible for three patients. Alongside their profession specific daily routine, they conduct daily rounds and handovers and plan, deliver and reflect on patient treatment together in an interprofessional team. The facilitators supervise their actions mainly passively and only interfere if necessary. They support the undergraduates by encouraging them to autonomous and collaborative decision making and problem solving, as well as by giving them feedback and facilitating reflective processes.

### Outcomes

Primary outcome are interprofessional teamwork and collaboration, interprofessional learning and interprofessional interaction as measured with the University of West of England Interprofessional Questionnaire (UWE-IP) [25]. Within a longitudinal comparison between T0, directly before the beginning of HIPSTA, T1, at the end of HIPSTA, and T2, 3 months after the end of HIPSTA, it will be assessed if participants develop more positive attitudes towards these interprofessional aspects due to their placement on the IPTW. Secondary outcomes are the differences in longitudinal comparison between T0, T1, and T2 for participants on the IPTW and on the comparison ward as measured with the “Interprofessional Socialization and Valuing Scale” (ISVS) [26] and the “Assessment of Interprofessional Team Collaboration Scale (AITCS)” [27]; as well as an in-depth insight in and a deeper understanding of the development of interprofessional competencies and beneficial and impeding factors for competency gain as assessed with the help of structured non-participant observations and video analysis of the handovers, focus groups and interviews.

### Study sample

All undergraduate participants and staff participants placed or employed on HIPSTA or its surrounding ward (intervention group), or on the comparison ward (comparison group) are included in the study and questioned to participate. To answer the research questions three different participant samples were identified and divided into three levels. Undergraduate participants on HIPSTA and the comparison ward are assigned to level one, staff working on the surrounding ward HIPSTA is integrated in and on the comparison ward is assigned to level two and the facilitators on HIPSTA are assigned to level three. All participants of the study have to be at least 18 years old.

For the medical undergraduates the placement on HIPSTA is part of their surgical four months placement during their practical year, after their study programme and before taking their final exams (sixth and final year of training). In Germany nursing training at present is predominantly not an academic programme. In most cases it is a hospital based 3 year vocational training programme in contrast to medical school programmes which are all university based [28]. Nurse undergraduates on HIPSTA are in their third year of training shortly before their final exams. The facilitators (level 3), nurses with an additional qualification as practical teachers and ward physicians, are prepared briefly beforehand for practical supervision on HIPSTA by members of the project team. This preparation encompasses information on interprofessional collaboration and on the facilitators’ tasks of enabling self-responsible working of the undergraduates and supporting them.

Within 15 months 12 consecutive cohorts comprising four medical and four nursing undergraduate participants at a time will be placed on HIPSTA for four weeks. In terms of the pragmatic approach of the study all of the undergraduates employed on HIPSTA and all undergraduates on the comparable ward are integrated in data collection following an exploratory design. By that a sufficient sample size for longitudinal analysis of all three points in time can be achieved, as a high drop-out rate is expected. Hence, an estimated number of about 96 undergraduate participants will be recruited (level 1) within the phase of one year (April 2017 – June 2018). Accordingly, all affected staff members working on the ward HIPSTA is integrated in, and all colleagues working on the comparable ward, as well as all facilitators employed on HIPSTA are recruited within the time of data collection (April 2017 – June 2018). The number of colleagues and facilitators that can be recruited for the study can roughly be projected at 30 to 60 participants, depending on the number of employees working on the wards at the points in time of the survey (levels 2 and 3).

### Data collection

Within a mixed methods design quantitative and qualitative data is collected. Staff and facilitators working on HIPSTA and staff working on the comparison ward are informed about the study before the first cohort’s placement on HIPSTA and their consent to participate is obtained. Undergraduates are informed about the study by the researchers during the introduction day and their consent to participate is obtained before starting their placement on HIPSTA or on the comparison ward. Besides being informed verbally, staff, facilitators and undergraduates receive an information sheet, which clarifies how their data is used and gives them the contact information on the principle investigator. Since May 2018, in an updated version according to the General Data Protection Regulation, the contact information of the data protection officers of the faculty and of the state is provided. They are informed that their participation in the study is voluntary and that they can withdraw their consent to participate any time. After being informed they can give their consent to participate by signing a consent form.

Quantitative data is collected at different points in time for undergraduate participants (level 1), staff participants (level 2) and facilitators (level 3), with a questionnaire consisting of the validated German version of the University of Western England Interprofessional Questionnaire (UWE-IP) [25], the German version of the Interprofessional Socialization and Valuing Scale (ISVS) [26] and the German version of the Assessment of Interprofessional Team Collaboration Scale (AITCS) [27]. The German versions of the ISVS and AITCS are subject to testing of psychometric properties.

Three scales of the UWE-IP, each consisting of nine items, are applied: The “Communication and Teamwork Scale” uses a four-point Likert-scale with ratings from 1 = strongly disagree to 4 = strongly agree. Both the “Interprofessional Learning Scale” and the “Interprofessional Interaction Scale” use a five-point Likert scale with ratings from 1 = strongly disagree to 5 = strongly agree. Scale scores are calculated by summing items, achieving scores from nine to 36 (communication and teamwork), and nine to 45 for the other two scales.

The ISVS comprises 21 items, each item beginning with *At this point in time, based on my participation in interprofessional education activities and/or clinical practice …,.* Items can be scored on a seven-point Likert scale with ratings from 1 = not at all to 7 = to a very great extend and a further “0= not applicable” option. Items are summed achieving scores from 0 to 147. The AITCS consists of 37 items, each with the introduction *When we are working as a team …*,. Items are scored on a five-point Likert scale with ratings from 1 = never to 5 = always, including a response option “no answer”. The AITCS consists of three subscales, namely “partnership” (19 items), “cooperation” (11 items) and “coordination” (7 items).

**Fig. 1:**
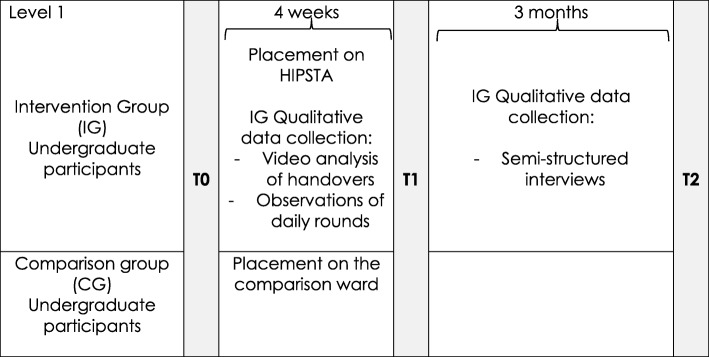
Data collection for undergraduate participants during their 4 week placement on HIPSTA. Points in time, when quantitative and qualitative data will be collected from undergraduate participants of each cohort placed on HIPSTA and on a comparable ward throughout four-weeks of placement and three months follow up

**Fig. 2:**
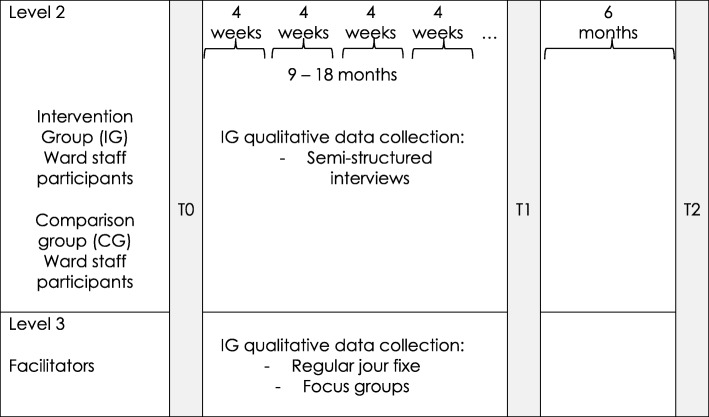
Data collection for staff participants and facilitators. Points in time, when quantitative and qualitative data will be collected from the staff employed on the ward, HIPSTA is integrated in and on the comparable ward as well as from the facilitators throughout one year of evaluation phase and six months follow up

All three questionnaires are administered to the undergraduate participants and the staff participants (medical and nursing) directly before the beginning of HIPSTA, at the end and as follow-up. Hence, all undergraduate participants of each cohort fill out the questionnaires at the beginning of their introduction day (T0), four weeks later, at the last day of their placement (T1) and three months after their placement (T2) (see Fig. 1), whereas the staff participants and the facilitators fill them out shortly before the general start of HIPSTA (T0), after about 12–18 months (T1), and six months later as follow up (T2) (see Fig. 2).

Qualitative data comprises audio recorded focus groups and interviews with the undergraduate participants of the intervention group at the end of their placement on HIPSTA and with the facilitators on HIPSTA, based on semi-structured guidelines. Two handovers per cohort are video recorded and two to four daily rounds per cohort are observed overtly by two non-participating researchers using a self-developed assessment sheet, containing items addressing the interprofessional collaboration and communication. Figures 1 and 2 show the schedule for data collection during the implementation of HIPSTA. The four weeks placement on HIPSTA includes the introduction day at the beginning, where study approval of the undergraduate participants is obtained.

### Data analysis

Quantitative data will be analysed descriptively (frequencies, mean, SD). Scale scores will be calculated, and differences between time points will be analysed for each of the questionnaires (UWE-IP, ISVS, AITCS) individually (primary outcome - UWE-IP scales) in a longitudinal comparison and tested with an analysis of variance (ANOVA). Data is analysed with focus on longitudinal and intergroup comparisons concerning cohort, profession and gender, as well as comparisons with the undergraduate and staff participants on the comparable ward without special interprofessional training (comparison group). Qualitative data will be analysed within qualitative content analysis deductively and inductively using the computer-assisted data analysis program MAXQDA in order to generate categories for hypothesizing [29]. Inductive analysis enables an in depth insight into participants’ experiences and behaviours. Quantitative data can be complemented. The deductive approach is chosen to cluster and compare data, especially in terms of a triangulation with the quantitative data in order to gain deeper insight from multiple perspectives in various chosen aspects.

## Discussion

A placement on an interprofessional training ward does not only address interprofessional competencies on a theoretical level but contributes to prepare undergraduate health professionals in a real-life clinical setting for future interprofessional collaborative practice in order to improve patient care. The four weeks placement on HIPSTA facilitates undergraduate health professionals’ competency and professional identity development, for example due to responsibility for the patients’ well-being, decision making processes with health care team members and patients, and thus clarification of the various roles in the team. The amount of responsibility undergraduate participants have on HIPSTA is unusual in undergraduate education for medical and nursing training in Germany [17, 30]. Furthermore, they normally do not have that many interprofessional encounters and especially do not focus on working together in interprofessional teams [2, 17, 31]. Undergraduate participants on HIPSTA can learn what it means to be responsible for patient care and what role interprofessional collaborative practice and communication play in this context whilst being supervised by facilitators. Not only interprofessional competencies can be developed but it is assumed that the placement on HIPSTA also affects the professional and interprofessional role clarification, due to freedom of decision, responsibility, intense patient contact, being part of an interprofessional team, participating in decision making processes and identifying working areas and interfaces (cf. [10, 18–20]). Thus undergraduate health professionals can learn with, about and from each other within a real life practice setting. Assessing the undergraduate participants’, staff participants’ and facilitators’ experiences can help to identify preconditions that have to be fulfilled for long-term implementation of an interprofessional training ward into the medical and nursing curricula.

### Trial status

The placement of the first cohort of undergraduates started on the 10th of April, 2017. At present, questionnaires have been collected from 112 undergraduate participants and 52 staff participants. 5 focus groups with 36 undergraduate participants and 4 focus groups with 9 facilitators, as well as individual interviews with 31 undergraduates and 9 facilitators have been conducted. To date 30 ward rounds and 14 handovers have been observed.

## Abbreviations

AITCS: Assessment of Interprofessional Team Collaboration Scale
ANOVA: Analysis of Variance
CG: Comparison Group
HIPSTA: Heidelberger Interprofessionelle Ausbildungsstation
IG: Intervention Group
IPCP: Interprofessional Collaborative Practice
IPE: Interprofessional Education
IPTW: Interprofessional Training Ward
ISVS: Interprofessional Socialization and Valuing Scale
SD: Standard Deviation
UWE-IP: University of Western England Interprofessional Questionnaire
